# Why Breeding Values Estimated Using Familial Data Should Not Be Used for Genome-Wide Association Studies

**DOI:** 10.1534/g3.113.008706

**Published:** 2013-12-20

**Authors:** Chinyere C. Ekine, Suzanne J. Rowe, Stephen C. Bishop, Dirk-Jan de Koning

**Affiliations:** * The Roslin Institute and R(D)SVS, University of Edinburgh, Easter Bush, Midlothian EH25 9RG, Scotland, United Kingdom; † Department of Animal Breeding and Genetics, Swedish University of Agricultural Sciences, 750 07 Uppsala, Sweden

**Keywords:** genome-wide association, family structure, type I error, statistical power

## Abstract

In animal breeding, the genetic potential of an animal is summarized as its estimated breeding value, which is derived from its own performance as well as the performance of related individuals. Here, we illustrate why estimated breeding values are not suitable as a phenotype for genome-wide association studies. We simulated human-type and pig-type pedigrees with a range of quantitative trait loci (QTL) effects (0.5–3% of phenotypic variance) and heritabilities (0.3−0.8). We analyzed 1000 replicates of each scenario with four models: (a) a full mixed model including a polygenic effect, (b) a regression analysis using the residual of a mixed model as a trait score (so called GRAMMAR approach), (c) a regression analysis using the estimated breeding value as a trait score, and (d) a regression analysis that uses the raw phenotype as a trait score. We show that using breeding values as a trait score gives very high false-positive rates (up 14% in human pedigrees and >60% in pig pedigrees). Simulations based on a real pedigree show that additional generations of pedigree increase the type I error. Including the family relationship as a random effect provides the greatest power to detect QTL while controlling for type I error at the desired level and providing the most accurate estimates of the QTL effect. Both the use of residuals and the use of breeding values result in deflated estimates of the QTL effect. We derive the contributions of QTL effects to the breeding value and residual and show how this affects the estimates.

Genome-wide association studies (GWAS) are now commonplace in humans, livestock, plants, and model organisms. A commonality among these studies is that genetic links exist between genotyped subjects and these must be accounted for in statistical analyses. Several approaches have been proposed to take account of these genetic structures in GWAS (Yu *et al.* 2006; Aulchenko *et al.* 2007a; Kang *et al.* 2008), resulting in a range of statistical tools such as TASSEL (Zhang *et al.* 2010), EMMA(X) (Kang *et al.* 2010) and GenABEL (Aulchenko *et al.* 2007b). Somewhat less attention has been paid to the definition of the trait value that is used for the GWAS.

In many situations, a phenotypic trait may be decomposed into an estimated breeding value (EBV) and a residual. The EBV is an estimated measure of the additive genetic merit of an individual (*e.g.*, animal, plant, tree) for the given trait based on its own performance and/or that of genetically related individuals. In the genome-wide rapid association using mixed model and regression (GRAMMAR) approach, the observed phenotype is analyzed under a mixed model resulting in an EBV and a residual, with the latter being used as the trait value for GWAS (Aulchenko *et al.* 2007a). Other investigators, however, have used the EBV of the individual as the trait score for GWAS, assuming that it encompasses the best estimate of the genetic merit of an individual (Johnston *et al.* 2011; Becker *et al.* 2013; Čepica *et al.* 2013). As a citation from Becker *et al.* (2013) shows, the EBV is sometimes considered the best estimate of the genetic merit of an individual:

Breeding values have the advantage that they are free of systematic environmental effects on measured phenotypes, as these effects are considered in the statistical model used for the estimation of EBVs. Additionally, they reflect the genetic makeup more accurately because they do not solely rely on own records but include information from all measured relatives.

We will show here with a straightforward simulation study that the “information from all measured relatives” is a prime source of false-positive results in GWAS. We note that this insight is neither profound nor novel, but our aim is to provide a clear and concise illustration that using EBV comprising familial information (*e.g.*, parents, sibs, etc.) can give much greater false-positive rates than ignoring family relationships altogether.

## Materials and Methods

### Simulations

The simulation scheme follows that of (Aulchenko *et al.*, 2007a) with two simulated family structures (human and pig) and one complex real pedigree (pig in this example). For the human pedigree, we simulated 337 nuclear families of three full-sibs with parents that are not related to each other or any of the other parents. For the pig pedigree, we simulated 10 sires, each mated to 10 dams that had 10 or 11 offspring, resulting in 1010 measured individuals for analysis. For the real pig pedigree, we randomly sampled 1010 last-generation offspring from a total pedigree of 5390 commercial pigs and included either two or five generations of pedigree information. The latter was to test the impact of the depth of pedigree information on performance of the EBV approach. The pedigrees that were the basis for the simulations are presented in Supporting Information, File S1. Each of the 46 scenarios was simulated in 1000 replicates using the MORGAN genedrop program (George *et al.* 2005). MORGAN genedrop simulates genotypes at marker loci, trait genotypes, and polygenic values contributing to the quantitative traits. Quantitative traits were defined as the sum of the single-nucleotide polymorphism (SNP) effect, the polygenic effects, and a random environmental error. Two SNP genotypes were simulated and analyzed for association: one SNP was not linked with the trait of interest, or any other marker, and used for studying the type I error rate. For studying power, a causal SNP with an additive effect of 4.0 and a minor allele frequency of 0.3 was simulated explaining 0.5, 1, 2, or 3% of the total variation in the trait. The simulated traits had a total heritability of 0.30, 0.40, 0.50, 0.60, and 0.80. The QTL effect and variance due to the QTL were constant throughout the simulations whereas the polygenic variance and the residual variance were scaled to achieve the different QTL contributions and overall heritabilities. An example of the MORGAN genedrop script files that were used for simulation is given as File S2.

### Statistical analyses

The simulated data were analyzed using four different approaches:

1. Measured genotype: The SNP to be tested for association was fitted as a covariate in a polygenic mixed model (1), which accounted for familial relatedness of individuals in the pedigree using the additive genetic relationships among individuals. The SNP effect and polygenic effect were estimated together using this model:
  y=μ+wa+Zu+e (1)
  where **y** is the vector of trait values, μ is the overall mean, a is the additive QTL effect, **u** and **e** are vectors of additive polygenic effects (random), and random residuals, respectively; **u** ~ N(0, ***A***σ^2^_a_), where ***A*** is the additive genetic relationship matrix based on pedigree information and **e** ~ N(0, **I**σ^2^_e_), where **I** is an identity matrix; σ^2^_a_ and σ^2^_e_ are the additive genetic and residual error variance, respectively. **w** is a vector of marker genotypes (codes as 0, 1, 2) and ***Z*** is an incidence matrix related to polygenic effects.
2. GRAMMAR: The GRAMMAR approach consists of two steps (Aulchenko *et al.* 2007a): the first step accounts for the familial dependence among family members and the second step tests the single SNP effect on the remaining variation by analysis of variance.
  Step 1: For the simulated trait score, we fitted the following mixed model [with the same variable definitions as (1)] without the marker effect:
    y=μ+Zu+e (2)
  Step 2: Using the estimated residuals from Step 1 as the new quantitative trait (y*), the marker genotype effect of each SNP on the trait was tested by linear regression:
    $$y^{*}=\mu +wa+e^{*}$$ (3)
3. Ignoring family structure (IF): The IF analysis is comparable with the second step of the GRAMMAR analysis. It uses a direct regression of the phenotypic observation (**y**) on the SNP data and does not take account of family relationships.
4. EBV: Similar to GRAMMAR but in this analysis the EBV from the polygenic model [û, from model (2)] is used as a trait score (y*) for the association study (3).

All analyses were performed in ASReml (Gilmour *et al.* 2009). The type I error of each scenario was estimated using the unlinked SNP and a tabulated threshold of *F* > 3.85 (*P* < 0.05). The statistical power to detect the causal SNP was estimated using either the tabulated *F*-threshold of 3.84 or an empirical threshold based on a 5% error rate for the unlinked SNP. In order to facilitate the computational load of 1000 replicates of 46 scenarios, the simulations and analyses were run on the Edinburgh Compute and Data Facility.

## Results

### Type I error

The false-positive rates (FPRs) for the four methods are summarized in Table 1, averaged across QTL effect size. Because they were estimated on unlinked QTL they were, as expected, observed to be independent of QTL size. The GRAMMAR approach was conservative, whereas the measured genotype approach performed very close to the tabulated threshold. Use of either the EBV or ignoring family relationships resulted in much greater levels of false-positive results. However, the FPR depended on the family structure in the data, with a greater degree of relatedness for the pig scenarios than the human scenarios. Particularly for the pig data, FPR for IF increased with increasing heritability whereas it decreased for EBV. Conversely, GRAMMAR was slightly less conservative for the human data than for the pig data.

**Table 1:** Type 1 error rate for MG, GRAMMAR, EBV, and IF analysis for the simulated human and pig population structures, averaged across QTL effects for each heritability (h^2^) class

h^2^	Human Population				Pig Population			
	MG	GRA	EBV	IF	MG	GRA	EBV	IF
30%	0.050	0.038	0.139	0.067	0.051	0.017	0.630	0.268
40%	0.047	0.031	0.127	0.068	0.057	0.018	0.600	0.324
50%	0.044	0.025	0.122	0.070	0.043	0.009	0.579	0.352
60%	0.055	0.031	0.144	0.091	0.054	0.012	0.570	0.401
80%	0.053	0.023	0.135	0.111	0.045	0.007	0.485	0.445

MG, measured genotype; GRAMMAR, genome-wide rapid association using mixed model and regression; EBV, estimated breeding value; IF, ignoring family; GRA, GRAMMAR.

The simulations based on the commercial pig pedigree also showed a large type I error when using EBV (Table S1), albeit slightly lower than for the simulated pedigree. This was due to the smaller family sizes in the sample from the real pedigree compared to the simulated pedigree. The use of five generations of pedigree in the mixed model (2) gave a greater FPR when we used EBV as the trait score compared to using only two generations (Table S1). The use of GRAMMAR was more conservative when applying five generations of pedigree compared with two. There was no clear trend in type I error when using the measured genotype approach (1) or ignoring family structure: in some scenarios using five generation of pedigree gave a more conservative type I error, whereas in other scenarios it was more liberal than using two generations of pedigree information (Table S1).

### Power

**Figure 1:**
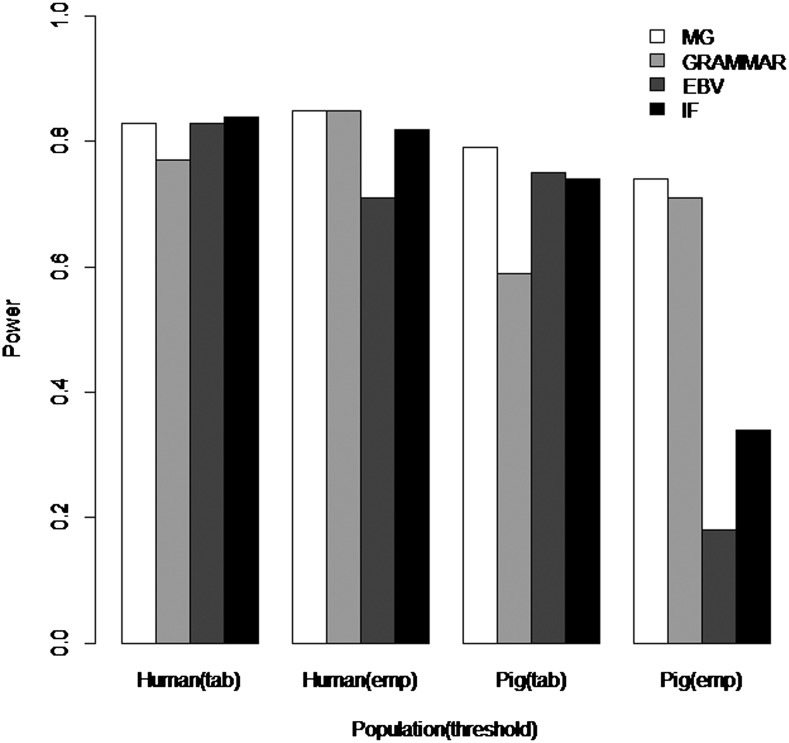
Empirical and tabulated power of detecting a QTL that explains 1% of phenotypic variance in a trait with 40% heritability. MG: measured genotype; tab: tabulated power, emp: empirical power.

The full comparisons of statistical power to detect the QTL across all scenarios are presented in Table S2 for the simulated human and pig pedigrees, and in Table S1 for the real pedigree simulations. Measured genotype analyses (1) usually, but not always, had the greatest power, irrespective of whether empirical or tabulated thresholds were used (Figure 1 and Table S2). Using EBV or ignoring family relationships had greater power when using tabulated (rather than empirical) thresholds (Figure 1 and Table S2), but at the cost of high FPRs (Table 1). GRAMMAR was conservative when using tabulated thresholds but comparable in power with using measured genotype when applying empirical thresholds (Figure 1 and Table S2), in agreement with (Aulchenko *et al.* 2007b).

For scenarios in which the heritability and QTL effect sizes were low, *e.g.*, a heritability of 30% and a QTL explaining 1% of phenotypic variance as shown in Figure 1, the human pedigree had greater power than the pig pedigree. However, at greater heritabilities, these differences diminished and for some scenarios the pig pedigree had the higher power (Table S2). For the simulations based on the commercial pig pedigree, the measured genotype approach had the highest power regardless of whether the thresholds were tabulated or based on empirical results (Table S1). The two-generation pedigree generally gave slightly greater power to detect QTL than the five-generation pedigree.

### Estimated effects

The estimates of the QTL effect and the empirical standard deviation more than 1000 replicates are summarized in Table 2. Noting that the expected effect size was 4.0, using measured genotype gave the most accurate, and apparently unbiased, estimates of the QTL effect regardless of variance explained by the QTL or overall heritability (Table 2). Ignoring family information also gave accurate estimates of the QTL effect, apart from when the proportion of variance explained by the QTL was small in which case, the estimates were slightly inflated (Table 2). When using GRAMMAR or EBV the QTL effects were underestimated dramatically. This confirms earlier observations that GRAMMAR underestimates the QTL effects (Aulchenko *et al.* 2007a; Crooks *et al.* 2009).

**Table 2:** Mean estimates (mean) and empirical standard deviations (SD) of QTL effect for different association analyses across a range of relative QTL effects and heritabilities (h^2^) in simulated human and pig pedigrees

h^2^		Human								Pig							
	QTL Effect	MG		GRAMMAR		EBV		IF		MG		GRAMMAR		EBV		IF	
		Mean	SD	Mean	SD	Mean	SD	Mean	SD	Mean	SD	Mean	SD	Mean	SD	Mean	SD
30%	0.5%	4.01	1.83	2.55	1.18	1.49	0.83	4.02	1.86	4.09	2.01	2.15	1.09	2.64	2.05	4.36	2.85
	1%	3.97	1.37	2.53	0.91	1.45	0.64	3.70	1.38	4.05	1.52	2.11	0.82	2.24	1.65	4.15	2.20
	2%	3.98	0.95	2.52	0.65	1.47	0.53	3.99	0.98	4.01	1.04	2.09	0.61	1.95	1.26	3.96	1.57
	3%	3.97	0.79	2.51	0.53	1.46	0.49	3.96	0.81	3.97	0.86	2.09	0.53	1.90	1.10	3.97	1.25
50%	0.5%	3.90	1.86	1.74	0.86	2.20	1.23	3.91	1.97	4.00	1.93	1.55	0.79	3.45	2.69	4.60	3.14
	1%	3.96	1.38	1.77	0.67	2.20	0.95	3.96	1.46	3.97	1.48	1.54	0.63	2.82	2.05	4.08	2.48
	2%	4.04	1.01	1.81	0.54	2.25	0.70	4.06	1.05	4.03	1.04	1.57	0.49	2.60	1.64	4.04	1.90
	3%	4.05	0.78	1.80	0.42	2.26	0.61	4.06	0.81	3.98	0.83	1.53	0.42	2.47	1.45	3.93	1.61
80%	0.5%	4.01	1.92	0.75	0.44	3.34	1.88	4.07	2.18	4.14	1.81	0.74	0.46	4.63	3.38	5.15	3.58
	1%	3.96	1.34	0.74	0.34	3.25	1.36	3.98	1.52	4.02	1.40	0.74	0.39	3.80	2.66	4.38	2.86
	2%	3.94	0.96	0.73	0.23	3.18	0.99	3.91	1.09	3.98	0.97	0.73	0.35	3.49	2.13	4.14	2.22
	3%	3.98	0.80	0.74	0.28	3.25	0.83	3.99	0.89	3.99	0.78	0.74	0.35	3.26	1.77	3.96	1.80

The simulated QTL effect was always 4. MG, measured genotype; GRAMMAR, genome-wide rapid association using mixed model and regression; EBV, estimated breeding value; IF, ignoring family.

A clear trend was apparent for the effect of the heritability on the estimates when using GRAMMAR or EBVs. With increasing heritability, the GRAMMAR estimates were increasingly biased downward while those from the EBV approach became less biased (Table 2). Furthermore, in the pig simulations the estimates of using EBV were more severely underestimated with increasing variance explained by the QTL. On the other hand, the estimates in which GRAMMAR was used appeared to be unaffected by the proportion of variance explained by the QTL in both the pig and the human simulations (Table 2). When we looked at individual replicates, it was apparent that the sum of the GRAMMAR and EBV estimates provided an unbiased estimate of the SNP effect. This could provide a quick estimate of the true effect of significant SNPs after GRAMMAR analyses, rather than re-estimating the effect in a full mixed model as suggested previously (Aulchenko *et al.* 2007a). Across all scenarios and analyses, the precision of the estimates increased as the proportion of variance explained by the QTL increased, as shown by the empirical standard errors (Table 2).

## Discussion

Our simulations have shown clearly that the use of EBV in association studies, incorporating information from relatives from the same or previous generations, can result in several problems, most notably huge increases in the type I error. This finding is attributed to the fact that when an individual’s EBV is estimated with familial information, the estimate is a linear combination of the individual’s phenotype, expressed as a deviation from the family mean, and the family mean itself. Although the individual phenotype captures the within-family segregation of the QTL, the family mean contains information on the QTL allele expressed by other family members. This “contamination” of the EBV by family information can affect both power and the FPR as follows. First, power can be reduced (as shown by results for the empirical thresholds in Table S1 and Table S2) as many sibs may have received alternative QTL alleles. This dilutes the SNP effect, and it will have greater impact in situations in which family information makes a greater contribution to the EBV. Second, there is a greater risk of FPRs, as any SNP that differs in frequency between families risks being correlated by chance with the family mean polygenic value for the trait and hence shows a significant association with the EBV. This finding implies that in analyses of real human pedigrees the type I errors are expected to be even more serious that those shown here in the simulated pedigrees as a result of minor ethnic variations from one pedigree to the next.

In the Appendix, we demonstrate how an EBV may be decomposed in individual and family information and into major gene and polygenic (unlinked) effects. With EBVs, it is apparent that the weighting given to the family mean is always greater than that given to the Mendelian sampling term, hence the risks of FPRs and reduced power described previously, with the converse true for residuals. The relative weightings, and the expected value of the regression of phenotype on marker, are slightly complex and depend on the trait heritability, the accuracy with which the family mean is estimated, and the QTL frequency. Derivations are shown in the Appendix for family information estimated solely from sib means; however, the same principles apply to information obtained from other sources. This is seen in Table S1, where the five-generation pedigree resulted in a greater FPR than the two-generation pedigree; because the family mean was estimated more accurately, a greater weighting was applied to the family mean resulting in a greater FPR.

In species in which the bulk of the information is derived from progeny testing, such as dairy cattle breeding, the contribution to the EBV coming from relatives other than the direct offspring becomes smaller and using EBV will be less detrimental than in the cases considered here. This is because offspring information directly estimates the Mendelian sampling term of the animal being evaluated, and hence assists in estimation of the QTL effect. We acknowledge that for many species, most commonly in dairy cattle where EBV are derived for bulls using a large number of daughter records, EBV for a wide range of traits are routinely available and are a convenient source of information. In these cases, the use of deregressed EBV has been proposed for GWAS and genomic prediction (Garrick *et al.* 2009). De-regressed EBV take account of the heterogeneous variances of EBV that are the result of, *e.g.*, different numbers of daughter records per sire. However, de-regressed EBVs do not remove the component of the EBV coming from information on other relatives. To remove effects from other relatives from the (deregressed) EBV, Garrick *et al.* (2009) suggested to adjust for the parent average effect. The use of de-regressed EBVs, adjusted for parent average effects, can be also relevant when the EBV is the result of repeated measurements that are not easily replaced by a single trait score for GWAS.

In summary, when each genotyped individual has its own associated trait score(s), we recommend the use of a measured genotype approach (1) or an approximation using GRAMMAR. Although GRAMMAR was once again shown to be conservative and give an underestimate of the QTL effect, recent developments of the GenABEL software have accounted for this in the GRAMMAR-Lambda module that provides an adjusted test static and a correction for the estimated QTL effect (Svishcheva *et al.* 2012). At all costs, naïve usage of EBVs incorporating familial information should be avoided, as use of EBVs will achieve the triple whammy of reducing power, increasing the FPR and misestimating QTL effect sizes.

## Supplementary Material

Supporting Information

## Appendix

### Decomposing the animal genotype

Assume that the phenotype for individual *i* is composed of a genotype and a residual (“environmental”) term. Thus:

$$P_{i}=G_{i}+E_{i}$$ (1)

Assuming that we are interested in the contribution of a major (additive) gene to the genotype, the individual’s genotype can be decomposed in several equivalent ways. First, the genotype can be decomposed into a polygenic component (uncorrelated with the major gene) and the direct additive effect of the major gene, *i.e.*:

$$G_{i}=G_{poly\_i}+g_{i}$$ (2)

Alternatively, the genotype can be decomposed into the family mean (*F*) plus a Mendelian sampling term (*M_i_*), ignoring the major gene effect for the time being. Thus;

$$G_{i}=F+M_{i}$$ (3)

Following the logic that the overall genotype can be decomposed into a family mean and a Mendelian sampling term, the same can be done with the polygenic terms, *i.e.*, *F_poly_* and *M_poly_*. Therefore:

$$G_{i}=F_{poly\_i}+M_{poly\_i}+g_{i}$$ (4)

Clearly, the true major gene effect will contribute to the individual’s deviation from the family mean, hence will be contained in the *Mi* term in equation 3. However, depending on the allele frequency of the major gene variant, the major gene effect will also contribute to the family mean. It is easily shown that if the major gene is additive with a frequency of the major allele *p*, and assuming Hardy-Weinberg equilibrium, then the expected value of the major gene contribution to the family mean is (*p* − *q*)*g*.

### Composition of EBV and residual

Now consider the situation in which we have phenotypic information on an individual and on *n* sibs, who have phenotypes *Ps*. Deeper pedigree is ignored. The EBV will be calculated as follows:

$$\begin{array}{l} EBV_{i}=b1.P_{i}+b2.\sum \frac{Ps}{n}\\ =b1\left(F+M_{i}+E_{i}\right)+b2\left(F+\sum \frac{Ms}{n}+\sum \frac{Es}{n}\right)\\ =\left(b1+b2\right)F+b1.M_{i}+\left(b1.E_{i}+b2\sum \frac{Ms}{n}+b2\sum \frac{Es}{n}\right) \end{array}$$ (5)

The first term of equation (5) contains data potentially leading to false positives, *i.e.*, the false-positive signal, as any SNP that differs in frequency between families and shows a correlation with the family mean may show up as a significant effect. Depending on allele frequency the family mean will also contain information on the major gene of interest, as well. The second term contains the true major gene effect, *i.e.*, the true signal. The third term contains noise that is uncorrelated with the major gene effect in individual *i* that potentially masks the signal.

The residual term is simply the phenotype for the individual minus the EBV:

$$\begin{array}{l} Resid=P_{i}-\left(b1.P_{i}+b2.\sum \frac{Ps}{n}\right)\\ =\left(1-b1-b2\right)F+\left(1-b1\right)M_{i}+\left(\left(1-b1\right)E_{i}-b2\sum \frac{Ms}{n}-b2\sum \frac{Es}{n}\right) \end{array}$$ (6)

Note that for the EBV, the weighting given to the family mean is always greater than that given to the Mendelian sampling term((b1+b2)>b1), hence a much greater risk of false positives as well as a masking of true effects, whereas the converse is true for the Residual.

### Calculating the weighting factors

The weighting factors *b*1 and *b*2 can easily be calculated from standard selection index theory, where ***Pb*** *=* ***Gv***. In this case with one trait the vector ***v*** is simply a unity scalar, ***P*** is the phenotypic variance/covariance matrix of the phenotypes, and ***G*** is the genetic covariance vector between the information sources and the individual’s true genotype.

If the phenotypic variance is set to one, *i.e.*, $\sigma_{p}^{2}=1$, and using standard expectations for variance and covariances, then $Cov\left(P_{i},\sum \frac{Ps}{n}\right)=Cov\left(G_{i},\sum \frac{Ps}{n}\right)=rh^{2}$ and

$Var\left(\sum \frac{Ps}{n}\right)=\left(1+\frac{r(n-1)h^{2}}{n}\right)$, where *r* is the coefficient of relationship between sibs, *e.g.*, 0.5 for full sibs. For the full-sib scenario and assuming no common environmental effects, after some algebra and removing the common factor $\left(h^{2}/\left[1+0.5\left(n-1\right)h^{2}-0.25nh^{4}\right]\right)$, we obtain the following relative weighting factors: $b^{*}1=1+0.5\left(n-1\right)h^{2}-0.25nh^{4}$ and $b^{*}2=0.5n\left(1-h^{2}\right)$, where * indicates that the terms have been scaled by the common factor. Therefore, the relative weights depend, as you would expect, on the trait heritability and the information available to calculate the family mean (*n*).

### Expectations of estimated genotype effects

Let the major gene effect be indicated by a SNP marker, and the regression of the trait on the allele count (*e.g.*, −1, 0, 1) for the marker be defined as *β*.

#### Estimating the genotype as a fixed covariate in mixed model equations:

Let the estimated *g* be *ĝ*. Invoking the BLUE properties of Mixed Model BLUP equations, for fixed effects E(*ĝ*) = *g*. Thus, the estimate of g is unbiased.

#### Estimating the genotype from regression of phenotype on markers, *ignoring families:*

Combining equations 1 and 2, we have

$$P_{i}=G_{poly\_i}+g_{i}+E_{i}$$

Therefore, the expected value of the regression equation is:

$$\begin{array}{l} E\left[\beta \left(G_{poly\_i}+g_{i}+E_{i},marker\right)\right]\\ =E\left[\beta \left(G_{poly\_i},marker\right)\right]+E\left[\beta \left(g_{i},marker\right)\right]+E\left[\beta \left(E_{i},marker\right)\right]\\ =0+g+0\\ =g \end{array}$$

Thus, the estimate of *g* is again unbiased.

#### Estimating the genotype from regression of EBV or residual on markers:

First note that by definition $P_{i}=EBV_{i}+Resid_{i}$. Therefore, if $\beta \left(P_{i},marker\right)$is an unbiased estimate of *g*, then the sum $\beta \left(EBV_{i},marker\right)+\beta \left(Resid_{i},marker\right)$is also an unbiased estimate of *g*.

Consider $\beta \left(EBV_{i},marker\right)$. As described previously, $EBV_{i}=b1.P_{i}+b2.\sum \frac{Ps}{n}$; therefore, we wish to find the expected value of the regression: $\beta \left(b1.P_{i}+b2.\sum \frac{Ps}{n},marker\right)$. The first term yields a contribution b1g to the expected regression coefficient. The second term is:

$b2.\beta \left(\sum_{}^{}\frac{Ps}{n},marker\right)=b2.\beta \left(\sum_{}^{}\frac{Gs_{poly}}{n}+\sum_{}^{}\frac{gs}{n}+\sum_{}^{}\frac{Es}{n},marker\right)$. Only $\beta \left(\sum_{}^{}\frac{gs}{n},marker\right)$ has a non-zero expectation; when *p*≠0.5, the individual and family mean genotype will be correlated. This regression term is simply (p−q)g.

Therefore:

E[β(EBV,marker)]=(b1+b2(p−q))g (7)

and consequently:

$$E\left[\beta \left(Resid_{i},marker\right)\right]=\left(1-b1-b2(p-q)\right)g$$ (8)

Therefore, the expected effect sizes when using EBVs or residuals are biased downward, with the bias being a function of the heritability, the quantity of familial information, and the frequency of the major gene.
