# Supplementary material for: Why Breeding Values Estimated Using Familial Data Should Not Be Used for Genome-Wide Association Studies
Source: G3 (Bethesda). 2013 Dec 20;4(2):341–7. doi: 10.1534/g3.113.008706 (PMC3931567; doi:10.1534/g3.113.008706)
Supplement: Supporting Information [file supp_4_2_341__index.html]

Why Breeding Values Estimated Using Familial Data Should Not Be Used for Genome-Wide Association Studies — Supporting Information 

# Why Breeding Values Estimated Using Familial Data Should Not Be Used for Genome-Wide Association Studies

## Supporting Information for Ekine *et al.*, 2014

**Files in this Data Supplement:**

- Supporting Information - Tables S1-S2 and Files S1-S2 (PDF, 323 KB)
- Table S1 - Type 1 error and power using tabulated and empirical thresholds for simulations based on a commercial pig pedigree using 2 or 5 generations of pedigree information. (PDF, 302 KB)
- Table S2 - Tabulated (Tab) and empirical (Emp) power for different association analyses across a range of relative QTL effects and heritabilities (h2) in simulated human and pig pedigrees. (PDF, 308 KB)
- File S1 - Excel file with all the different pedigrees that were used for simulation (.xlsx, 254 KB)
- File S2 - An example of the script file that was used to run the genedrop simulation within MORGAN (.txt, 1 KB)
