# Supplementary material for: Why Breeding Values Estimated Using Familial Data Should Not Be Used for Genome-Wide Association Studies
Source: G3 (Bethesda). 2013 Dec 20;4(2):341–7. doi: 10.1534/g3.113.008706 (PMC3931567; doi:10.1534/g3.113.008706)
Supplement: Supporting Information [file supp_g3.113.008706_008706SI.pdf]

G3 · Genes | Genomes | Genetics

Why breeding values estimated using familial data should not be used for Genome-Wide Association Studies

C. C. Ekine[1, 3], S.J. Rowe[1,4], S.C. Bishop[1] and D.J. de Koning[1,2]

[1]The Roslin Institute and R(D)SVS, University of Edinburgh, Easter Bush, Midlothian EH25 9RG, Scotland, U.K.
[2]Department of Animal Breeding and Genetics, Swedish University of Agricultural Sciences, 750 07 Uppsala, Sweden.
[3]Present address: Livestock Gentec-AFNS, University of Alberta, 1400 College Plaza, 8215 – 112 Street, Edmonton, Alberta, T6G 2C8, Canada.
[4]Present address: AgResearch, Department of Reproduction and Genomics. Invermay Agricultural Centre, Private Bag 50034, Puddle Alley, Mosgiel 9053, New Zealand.

Correspondence: Dirk Jan de Koning
Department of Animal Breeding and Genetics
Swedish University of Agricultural Sciences
Box 7023, 750 07 Uppsala, Sweden
Phone: +46 18 672039
dj.de-koning@slu.se

DOI: 10.1534/g3.113.008706

Table S1 Type 1 error and power using tabulated and empirical thresholds for simulations based on a commercial pig pedigree using 2 or 5 generations of pedigree information. The simulated QTL explains 1% of phenotypic variance.

	Method of analysis											
heritability	MG[1]			GRAMMAR			EBV[2]			IF[3]		
	Tab[4]	FP[5]	Emp[6]	Tab	FP	Emp	Tab	FP	Emp	Tab	FP	Emp
	5-generation pedigree											
30%	0.80	0.05	0.81	0.70	0.02	0.81	0.79	0.42	0.42	0.80	0.11	0.67
40%	0.79	0.05	0.80	0.66	0.02	0.80	0.78	0.40	0.34	0.80	0.17	0.59
50%	0.80	0.05	0.80	0.68	0.02	0.80	0.75	0.36	0.33	0.79	0.18	0.56
60%	0.80	0.06	0.78	0.66	0.02	0.78	0.77	0.37	0.33	0.79	0.23	0.53
80%	0.83	0.04	0.84	0.70	0.01	0.84	0.73	0.31	0.37	0.75	0.25	0.45
	2-generation pedigree											
30%	0.85	0.06	0.82	0.76	0.03	0.81	0.82	0.35	0.40	0.85	0.13	0.72
40%	0.84	0.06	0.82	0.74	0.02	0.82	0.79	0.32	0.45	0.82	0.13	0.68
50%	0.82	0.05	0.80	0.70	0.01	0.83	0.79	0.32	0.44	0.82	0.18	0.63
60%	0.82	0.06	0.79	0.69	0.02	0.81	0.74	0.32	0.40	0.77	0.19	0.55
80%	0.84	0.05	0.85	0.71	0.02	0.83	0.76	0.28	0.45	0.79	0.23	0.54

[1]MG: Measured Genotype. [2]GRAMMAR: Genome-wide Rapid Association using Mixed Model and Regression [3]EBV: Estimated Breeding Value. [4]IF: Ignoring Family. [5]Tab: Power using tabulated threshold. [6]Emp: Empirical power using empirical threshold derived from the unlinked SNP. [7]FP: False positive rate using tabulated threshold.

Table S2 Tabulated (Tab) and empirical (Emp) power for different association analyses across a range of relative QTL effects and heritabilities (h^2) in simulated human and pig pedigrees.

		Human								Pig							
h^2	QTL effect	MG[1]		GRAMMAR[2]		EBV[3]		IF[4]		MG		GRA		EBV		IF	
		Tab[5]	Emp[6]	Tab	Emp	Tab	Emp	Tab	Emp	Tab	Emp	Tab	Emp	Tab	Emp	Tab	Emp
30%	0.5%	0.53	0.56	0.47	0.55	0.63	0.49	0.57	0.57	0.48	0.49	0.32	0.47	0.70	0.10	0.55	0.21
	1%	0.82	0.82	0.77	0.81	0.86	0.69	0.84	0.82	0.78	0.78	0.65	0.77	0.75	0.21	0.74	0.47
	2%	0.98	0.98	0.98	0.98	0.98	0.95	0.99	0.98	0.98	0.98	0.90	0.98	0.86	0.28	0.92	0.65
	3%	1.00	1.00	1.00	1.00	1.00	0.99	1.00	1.00	1.00	1.00	0.98	1.00	0.91	0.44	0.98	0.89
40%	0.5%	0.57	0.59	0.50	0.56	0.64	0.47	0.60	0.55	0.52	0.51	0.33	0.51	0.70	0.10	0.60	0.21
	1%	0.83	0.85	0.77	0.85	0.83	0.71	0.84	0.82	0.79	0.74	0.59	0.71	0.75	0.18	0.74	0.34
	2%	0.98	0.97	0.97	0.98	0.98	0.94	0.98	0.97	0.96	0.96	0.89	0.96	0.84	0.27	0.91	0.60
50%	0.5%	0.51	0.54	0.41	0.52	0.57	0.39	0.54	0.45	0.50	0.52	0.28	0.48	0.67	0.09	0.56	0.16
	1%	0.83	0.84	0.74	0.83	0.82	0.72	0.84	0.79	0.76	0.78	0.58	0.76	0.72	0.18	0.70	0.30
	2%	0.99	0.99	0.97	0.99	0.98	0.94	0.98	0.97	1.00	1.00	0.90	0.97	0.86	0.31	0.87	0.56
	3%	1.00	1.00	1.00	1.00	1.00	1.00	1.00	1.00	1.00	1.00	0.98	0.99	0.88	0.37	0.94	0.98
60%	0.5%	0.51	0.47	0.39	0.46	0.56	0.36	0.54	0.41	0.52	0.52	0.30	0.51	0.67	0.10	0.60	0.14
	1%	0.82	0.81	0.72	0.82	0.82	0.68	0.83	0.74	0.80	0.78	0.60	0.77	0.73	0.17	0.73	0.26
	2%	0.99	0.98	0.97	0.98	0.98	0.94	0.98	0.97	0.97	0.97	0.09	0.96	0.81	0.28	0.87	0.52
80%	0.5%	0.53	0.54	0.39	0.54	0.56	0.41	0.55	0.46	0.58	0.60	0.33	0.60	0.65	0.11	0.62	0.14
	1%	0.84	0.81	0.73	0.80	0.81	0.69	0.82	0.69	0.82	0.82	0.65	0.80	0.70	0.20	0.69	0.27
	2%	0.98	0.98	0.95	0.95	0.96	0.92	0.97	0.95	0.98	0.98	0.92	0.98	0.82	0.29	0.85	0.41
	3%	1.00	1.00	0.99	1.00	0.99	0.98	1.00	0.99	1.00	1.00	0.98	1.00	0.90	0.42	0.91	0.57

[1]MG: Measured Genotype. [2]GRAMMAR: Genome-wide Rapid Association using Mixed Model and Regression [3]EBV: Estimated Breeding Value. [4]IF: Ignoring Family. [5]Tab: Power using tabulated threshold. [6]Emp: Empirical power using empirical threshold derived from the unlinked SNP.

Files S1-S2

Available for download at http://www.g3journal.org/lookup/suppl/doi:10.1534/g3.113.008706/-/DC1

File S1 Excel file with all the different pedigrees that were used for simulation

File S2 An example of the script file that was used to run the genedrop simulation within MORGAN
