# Supplementary material for: Why Breeding Values Estimated Using Familial Data Should Not Be Used for Genome-Wide Association Studies
Source: G3 (Bethesda). 2013 Dec 20;4(2):341–7. doi: 10.1534/g3.113.008706 (PMC3931567; doi:10.1534/g3.113.008706)
Supplement: Supporting Information [file supp_g3.113.008706_TableS1.pdf]

Table S1 Type 1 error and power using tabulated and empirical thresholds for simulations based on a commercial pig pedigree using 2 or 5 generations of pedigree information.

The simulated QTL explains 1% of phenotypic variance.

	Method of analysis											
heritability	MG[1]			GRAMMAR			EBV[2]			IF[3]		
	Tab[4]	FP[5]	Emp[6]	Tab	FP	Emp	Tab	FP	Emp	Tab	FP	Emp
5-generation pedigree												
30%	0.80	0.05	0.81	0.70	0.02	0.81	0.79	0.42	0.42	0.80	0.11	0.67
40%	0.79	0.05	0.80	0.66	0.02	0.80	0.78	0.40	0.34	0.80	0.17	0.59
50%	0.80	0.05	0.80	0.68	0.02	0.80	0.75	0.36	0.33	0.79	0.18	0.56
60%	0.80	0.06	0.78	0.66	0.02	0.78	0.77	0.37	0.33	0.79	0.23	0.53
80%	0.83	0.04	0.84	0.70	0.01	0.84	0.73	0.31	0.37	0.75	0.25	0.45
2-generation pedigree												
30%	0.85	0.06	0.82	0.76	0.03	0.81	0.82	0.35	0.40	0.85	0.13	0.72
40%	0.84	0.06	0.82	0.74	0.02	0.82	0.79	0.32	0.45	0.82	0.13	0.68
50%	0.82	0.05	0.80	0.70	0.01	0.83	0.79	0.32	0.44	0.82	0.18	0.63
60%	0.82	0.06	0.79	0.69	0.02	0.81	0.74	0.32	0.40	0.77	0.19	0.55
80%	0.84	0.05	0.85	0.71	0.02	0.83	0.76	0.28	0.45	0.79	0.23	0.54

[1]MG: Measured Genotype. [2]GRAMMAR: Genome-wide Rapid Association using Mixed Model and Regression [3]EBV: Estimated Breeding Value. [4]IF: Ignoring Family. [5]Tab: Power using tabulated threshold. [6]Emp: Empirical power using empirical threshold derived from the unlinked SNP. [7]FP: False positive rate using tabulated threshold.
