# Supplementary material for: Why Breeding Values Estimated Using Familial Data Should Not Be Used for Genome-Wide Association Studies
Source: G3 (Bethesda). 2013 Dec 20;4(2):341–7. doi: 10.1534/g3.113.008706 (PMC3931567; doi:10.1534/g3.113.008706)
Supplement: Supporting Information [file supp_g3.113.008706_TableS2.pdf]

Table S2 Tabulated (Tab) and empirical (Emp) power for different association analyses across a range of relative QTL effects and heritabilities (h^2) in simulated human and pig pedigrees.

		Human								Pig							
h^2	QTL effect	MG[1]		GRAMMAR[2]		EBV[3]		IF[4]		MG		GRA		EBV		IF	
		Tab[5]	Emp[6]	Tab	Emp	Tab	Emp	Tab	Emp	Tab	Emp	Tab	Emp	Tab	Emp	Tab	Emp
30%	0.5%	0.53	0.56	0.47	0.55	0.63	0.49	0.57	0.57	0.48	0.49	0.32	0.47	0.70	0.10	0.55	0.21
	1%	0.82	0.82	0.77	0.81	0.86	0.69	0.84	0.82	0.78	0.78	0.65	0.77	0.75	0.21	0.74	0.47
	2%	0.98	0.98	0.98	0.98	0.98	0.95	0.99	0.98	0.98	0.98	0.90	0.98	0.86	0.28	0.92	0.65
	3%	1.00	1.00	1.00	1.00	1.00	0.99	1.00	1.00	1.00	1.00	0.98	1.00	0.91	0.44	0.98	0.89
40%	0.5%	0.57	0.59	0.50	0.56	0.64	0.47	0.60	0.55	0.52	0.51	0.33	0.51	0.70	0.10	0.60	0.21
	1%	0.83	0.85	0.77	0.85	0.83	0.71	0.84	0.82	0.79	0.74	0.59	0.71	0.75	0.18	0.74	0.34
	2%	0.98	0.97	0.97	0.98	0.98	0.94	0.98	0.97	0.96	0.96	0.89	0.96	0.84	0.27	0.91	0.60
50%	0.5%	0.51	0.54	0.41	0.52	0.57	0.39	0.54	0.45	0.50	0.52	0.28	0.48	0.67	0.09	0.56	0.16
	1%	0.83	0.84	0.74	0.83	0.82	0.72	0.84	0.79	0.76	0.78	0.58	0.76	0.72	0.18	0.70	0.30
	2%	0.99	0.99	0.97	0.99	0.98	0.94	0.98	0.97	1.00	1.00	0.90	0.97	0.86	0.31	0.87	0.56
	3%	1.00	1.00	1.00	1.00	1.00	1.00	1.00	1.00	1.00	1.00	0.98	0.99	0.88	0.37	0.94	0.98
60%	0.5%	0.51	0.47	0.39	0.46	0.56	0.36	0.54	0.41	0.52	0.52	0.30	0.51	0.67	0.10	0.60	0.14
	1%	0.82	0.81	0.72	0.82	0.82	0.68	0.83	0.74	0.80	0.78	0.60	0.77	0.73	0.17	0.73	0.26
	2%	0.99	0.98	0.97	0.98	0.98	0.94	0.98	0.97	0.97	0.97	0.09	0.96	0.81	0.28	0.87	0.52
80%	0.5%	0.53	0.54	0.39	0.54	0.56	0.41	0.55	0.46	0.58	0.60	0.33	0.60	0.65	0.11	0.62	0.14
	1%	0.84	0.81	0.73	0.80	0.81	0.69	0.82	0.69	0.82	0.82	0.65	0.80	0.70	0.20	0.69	0.27
	2%	0.98	0.98	0.95	0.95	0.96	0.92	0.97	0.95	0.98	0.98	0.92	0.98	0.82	0.29	0.85	0.41
	3%	1.00	1.00	0.99	1.00	0.99	0.98	1.00	0.99	1.00	1.00	0.98	1.00	0.90	0.42	0.91	0.57

[1]MG: Measured Genotype. [2]GRAMMAR: Genome-wide Rapid Association using Mixed Model and Regression [3]EBV: Estimated Breeding Value. [4]IF: Ignoring Family. [5]Tab: Power using tabulated threshold. [6]Emp: Empirical power using empirical threshold derived from the unlinked SNP.
